# Effectiveness of mindfulness-based interventions on empathy: A meta-analysis

**DOI:** 10.3389/fpsyg.2022.992575

**Published:** 2022-10-20

**Authors:** Zhengyu Hu, Yurong Wen, Yafei Wang, Yangyang Lin, Jian Shi, Zihan Yu, Youtian Lin, Yuling Wang

**Affiliations:** ^1^Department of Sport Rehabilitation, Shenyang Sport University, Shenyang, China; ^2^Rehabilitation Medicine Center, The Sixth Affiliated Hospital of Sun Yat-sen University, Guangzhou, China; ^3^Department of Sport Rehabilitation, Shanghai University of Sport, Shanghai, China; ^4^Postgraduate Research Institute, Guangzhou Sport University, Guangzhou, China

**Keywords:** mindfulness, empathy, meditation, randomized controlled trial, meta-analysis

## Abstract

Empathy is essential for human survival and social interaction. Although mindfulness-based interventions (MBIs) have been used to improve empathy in healthy populations, its therapeutic efficacy remains unknown. This study aims to investigate the therapeutic effects of MBIs on empathy in a healthy population and the potential factors affecting the efficacy of MBIs. The literature search focused on PubMed, Embase, Web of Science, Cochrane Library, and CNKI from inception to September 2022. Randomized controlled trials and quasi-experimental studies reporting the effects of using MBIs on empathy in healthy populations were included. A total of 13 studies were included in this review. Results of the meta-analysis showed that MBIs improved empathy (SMD, 0.372, 95% CI, 0.164–0.579, *p* = 0.001) in the healthy population compared with that in the control group. Moreover, results of the subgroup analysis showed that intervention dose (over 24 h vs. under 24 h), format (online vs. offline), and types (different types) were important factors affecting treatment outcomes. This comprehensive review suggests that MBIs are effective treatment for empathy in healthy population. Future research should markedly focus on large-sample, rigorously designed experiments to explore the long-term effects of MBIs on empathy and to elucidate the underlying mechanisms of MBIs. This study provides a reference for the daily application of MBIs.

## Introduction

Empathy is the ability to share and understand the inner state of others, enabling us to care for them, share knowledge, and work together to achieve goals (Preston and De Waal, 2017). Empathy is essential for understanding the cognitive and emotional processes of others in social settings (Keuken et al., 2011; Krall et al., 2016; Yang et al., 2018). In particular, empathy enhances emotional well-being (Morelli et al., 2015), greater social relationships (Morelli et al., 2017), and better social health (Zaki, 2020). Empathy also facilitates helping behavior, cooperation, and altruism (Feldmanhall et al., 2015). Impaired empathy is manifested by lacking understanding of the pain and plight of others, difficulty to be impressed, and appearing indifferent in real life, which is common in many psychiatric disorders (Decety and Moriguchi, 2007; Bragado-Jimenez and Taylor, 2012; Schreiter et al., 2013) and chronic pain (Sohn et al., 2016; De Tommaso et al., 2019; Ma et al., 2020; Mu et al., 2021; Zhang et al., 2021).

Although the general population around us are in good physical health, the empathy might be impaired for various reasons, such as stress, workplace violence, and lack of interpersonal interaction. Park et al. (2015) evaluated the relationships between stress and empathy, and found that the two were negatively correlated. Too much stress may be damaging empathy. The overuse of smart technology has led to a lack of interpersonal interaction among college students, and their empathy has declined significantly over the past decades (Konrath et al., 2011). Impaired empathy leads to be indifferent and difficulty in being moved in real life, further affecting normal daily interactions. The empathy of residents and nurses has also been impaired owing to the specificity of their professions. When confronted with the same pain stimulus, physicians rated pain intensity lower, but instead evoked higher emotional stress and empathic fatigue (Gleichgerrcht and Decety, 2014). A meta-analysis study showed that the empathy of clinical nurses also declined over time (Yi et al., 2021). Not only physicians and nurses, but also medical students showed a changing trend in pain empathy, with a significant decrease in empathy as the grade level increased (Neumann et al., 2011; Youssef et al., 2014; Wang et al., 2019). The impaired empathy of residents and nurses may lead to a lack of effective communication between them and their patients, thereby possibly leading to doctor-patient disputes and conflicts. Hence, we need an economical and efficient way to help people with poor empathy or prevent empathy from being damaged.

Mindfulness is a process of consciously engaging in the experience of the present moment (Creswell, 2017), and cultivating awareness of the present moment may help to change deeply ingrained cognitive patterns (Davis and Thompson, 2013). In recent years, mindfulness-based interventions (MBIs) have attracted considerable interest as a safe and effective integrative treatment. Consequently, there has been a significant increase in the number of randomized controlled trial studies on MBIs (Creswell, 2017). MBIs have multiple forms of intervention, such as mindfulness-based stress reduction (MBSR), mindfulness-based cognitive therapy (MBCT), mindfulness-based relapse prevention (MBRP), Internet and smartphone application mindfulness interventions, and other techniques incorporating mindfulness training (e.g., mindfulness meditation training, dialectical behavior therapy, and integrative body-mind training) (Creswell, 2017). MBIs have been used as a treatment for improving empathy (Can Gür and Yilmaz, 2020; Chen et al., 2021; Orosa-Duarte et al., 2021) and the results are usually positive.

In recent years, there has been a growing trend of meta-analyses in empathy, and most articles have discussed changes in empathy in certain diseases (Coundouris et al., 2020; Vucurovic et al., 2020; Pittelkow et al., 2021; Wright et al., 2021) or particular occupational groups (Abramson et al., 2020; Costa-Drolon et al., 2021; Yi et al., 2021). The evidence above has shown that empathy is impaired in many populations, but meta-analysis studies on how to improve empathy are limited. Some meta-analyses have proposed the use of non-invasive brain stimulation to improve empathy, but it is not convenient enough and is difficult to promote in the general population (Yang et al., 2018; Bahji et al., 2021). Several scholars have demonstrated the effectiveness of meditation for empathy improvement through meta-analysis. Luberto et al. (2018) explored whether or not meditation practices can be used to cultivate prosocial outcomes. The results identified were compared with the blank control or wait-list group, and improvements of empathy, compassion, and prosocial behaviors after meditation intervention are small to moderate. Another research by Kreplin et al. (2018) examining meditation interventions on prosociality in healthy adults has shown that the effect of meditation intervention on compassion and empathy is significant. Mindfulness-based interventions (MBIs) are important part of meditation. Mindfulness-based practices are widely found to have several benefits, including reducing stress, anxiety, and depression, and improving attentional focus, interpersonal relationships, and well-being (Astin, 1997; Brown and Ryan, 2003; Davidson et al., 2003; Kersemaekers et al., 2018; Slutsky et al., 2019). Fox et al. (2014) found that mindfulness meditation may lead to changes in brain structure and function related to emotion regulation, attention, and memory. Empathy might be improved in this way.

To the best of our knowledge, no studies have conducted a meta-analysis of the effect of MBIs on the improvement of empathic abilities. Thus, this study aimed to verify the effects of MBIs on empathy in general populations. Moreover, we explored whether or not the effects of the intervention were related to intervention dose, intervention form, intervention type, occupation, and family practice. We hypothesized that MBIs would have a larger pooled effect on empathy than the control group.

## Materials and methods

### Protocol and registration

The protocol was registered on PROSPERO (https://www.crd.york.ac.uk/prospero/) with registration number CRD42022315762. This meta-analysis was reported in line with the PRISMA guidelines and are shown in Supplementary material 1.

### Literature search

To identify articles for inclusion in the quantitative analysis, we searched the following databases for relevant studies from the initial availability date to September 2022: (1) Web of Science, (2) EMBASE, (3) Cochrane Library, (4) PubMed and (5) CNKI. The following keywords were searched: “empathy,” “mindfulness,” and “meditation.” No language restrictions were imposed. The search strategy is described in the Supplementary materials 2.

### Eligibility criteria

An initial screening of the articles retrieved from the electronic databases was first performed independently by two individuals (i.e., Z-YH and Y-RW). After removing duplicates, titles and abstracts were screened, and articles that did not meet the inclusion criteria were excluded. If relevance was not clear, the full text was read. After screening by two authors, the results will be checked and articles with discrepancies will be resolved through discussion. Agreement of included studies before reaching consensus can be quantified by using kappa statistics (Orwin and Vevea, 2009). If the consensus can't be reached, then the corresponding author will decide whether or not the article meets the inclusion criteria. Detailed inclusion criteria are as follows.

1) Types of participants: Healthy adults (over 18 years), any gender; children or adolescent were excluded; and disease or animal studies were excluded.
2) Types of studies: Parallel or crossover randomized controlled trials (RCTs) or quasi-experimental design.
3) Types of interventions: Mindfulness-based interventions of any type (e.g., MBSR, MBCT).
4) Types of outcome measures: Outcome measures included empathy.
5) Control group: No intervention or waiting list.
6) Studies must contain raw data of interest outcomes or can be extracted from figures and tables.

Exclusion criteria are as follows:

1) Studies published in the form of conference abstracts, case reports, and books.

### Data collection

Data extraction for each selected study was completed independently by two evaluators (i.e., Y-TL and JS), and reviewed and revised thereafter by the corresponding author. If the RCTs contained more than two arms, then we collected data from the separate treatment arms. A standard information extraction form was jointly designed by the two evaluators and contained the following aspects: (1) basic information: author, publication year, country of origin; (2) basic characteristics of subjects: occupation, gender, age, sample size, compliance, results; (3) study design: RCTs or quasi-experimental design; (4) intervention and control group parameters: intervention type and format, duration, frequency, and home practice; and (5) outcome assessment: empathy on the baseline and post-therapy with the outcome variables.

If the outcome was expressed only as a graph, then the software Engauge Digitizer 10.8 (Mitchell et al., 2017) (http://markummitchell.github.io/engauge-digitizer/) was used to extract the required data. When the raw data cannot be sufficiently extracted, we contacted the authors of the studies to provide them. RevMan 5.3 calculator was used to convert data to means and SDs when standard errors (SEs), confidence intervals (CIs), or IQRs were supplied rather than means and SDs.

### Risk of bias

The methodological quality of each included study will be determined by two authors (i.e., Y-FW and Z-HY) using the Cochrane Risk of Bias tool (Higgins Jpt, 2011). Factors assessed are as follows: (1) random sequence generation; (2) allocation concealment; (3) blinding of participants, outcome assessors, and investigators; (4) incomplete outcome information; (5) selective outcome reporting; and (6) other potential sources of bias. The studies will be judged as low, high, and unclear risk bias based on these factors. Before reaching the consensus, agreement of risk bias will be quantified by kappa statistics (Orwin and Vevea, 2009). Then, the two assessors will reach a consensus through discussion if they have any differences for the risk of bias of these studies. When a consensus cannot be reached between the two evaluators, the corresponding author will give his opinion and consensus of the majority will be adopted.

### Data synthesis and analysis

Meta-analysis was performed using Stata v17.0 software (StataCorp, Texas, US) with the metan command. We applied standardized mean differences (SMD) and 95% confidence intervals (CI) to evaluate the effect of MBIs on empathy outcome. Moreover, 95% CI was used to assess dichotomous variables, and *p* < 0.05 was considered a statistically significant difference. I^2^ statistic (Higgins et al., 2003) was used to assess whether or not heterogeneity exists between studies. If a high degree of heterogeneity was observed (e.g., *I*^2^ > 75%), then meta-analysis will no longer be performed. Sensitivity analyses will be performed when appropriate to investigate the reasons for heterogeneity. Fixed and random effects models will be used if *I*^2^ <50% and *I*^2^ > 50%, respectively. We assessed potential publication bias using funnel plots, and the extent of asymmetry was assessed quantitatively using Egger's test. Asymmetry of the funnel plot was adjusted for using metatrim.

We also analyzed five subgroups to explore the factors influencing the efficacy of MBIs on empathy: intervention dose (over 24 h vs. <24 h), format of intervention (online vs. offline), type of intervention (different types), occupation (non-medical related occupations vs. medical related occupations), and home practice (home practice vs. no home practice).

## Results

### Search results

**Figure 1:**
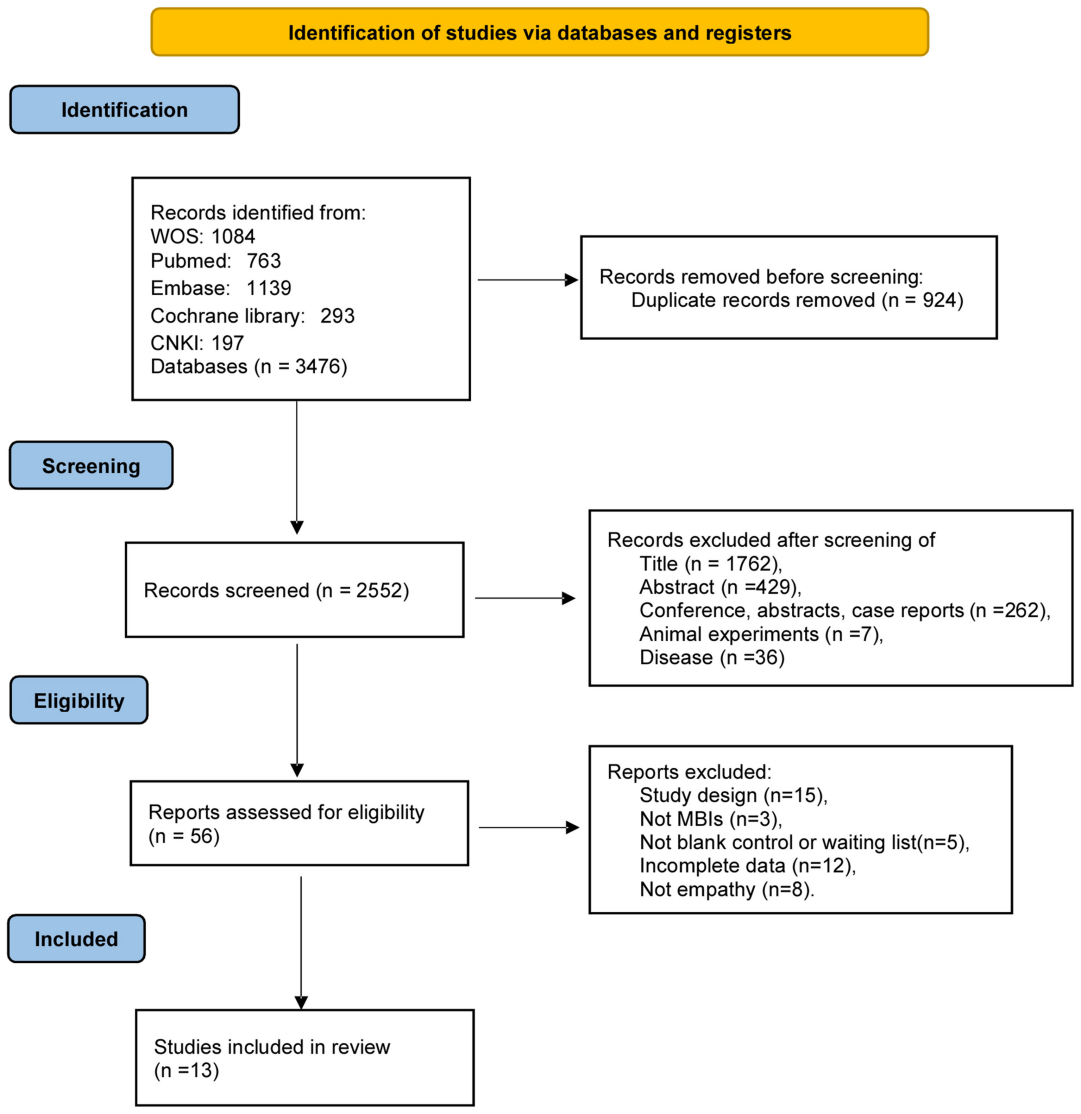
PRISMA flow chart of study selection.

We conducted a literature search and filtering according to the PRISMA guidelines, as detailed in Figure 1. In the preliminary search results, 3,476 articles were retrieved, 924 duplicate articles were removed, and 2,552 articles were removed by title, abstract, and other reasons that did not meet the standard criteria of this review. Thereafter, by evaluating the full text of the excess 56 articles, we excluded 43 studies for several reasons: study design (*n* = 15), not MBIs (*n* = 3), not blank control or waiting list (*n* = 5), incomplete data (*n* = 12), and not empathy (*n* = 8). The current study included 13 studies (15 data) in the meta-analysis.

### Study characteristics

We summarized the basic characteristics of the included articles (Tables 1, 2). The included studies were published between 1998 and 2021, with 9 out of 13 studies published in the past 5 years. Three articles each were published in Spain and the US, two in China, and one each in Turkey, Philippines, Netherlands, Sweden, and Canada.

**Table 1:** Characteristics of the included studies.

**Reference**	**Country**	**Sample**	**Mean Age**	**Gender (M/F)**	**Interventions**	**Control group**	**Intervention dose**	**Home practice**	**Compliance**	**Outcome variables**	**Results**
Asuero et al., 2014	Spain	*N =* 78; health care professionals	M_age_ = 47, SD=8.0	6/62	Mindfulness-based psychoeducational intervention	Waiting list	2.5 h once/week × 8 weeks and an intensive 8-hour session	no	78/68	JSPE	+
Can Gür and Yilmaz, 2020	Turkey	*N =* 154; undergraduate nursing students	M_age_ = 21.08, SD = 2.18	86/37	MBET	Blank control	1 h twice/week × 8 weeks	yes	154/123	JSPE	+
Centeno, 2020	Filipino	*N =* 30; Psychology Majors	M_age_ = 20.4, SD = 0.9	unclear	MBCT	Blank control	2 h twice/week × 8 weeks	yes	30/30	IRI	+
Chan et al., 2021	China	*N =* 60; undergraduate counseling trainees	unclear	20/30	MBCT	Waiting list	2 h twice/week × 8 weeks	yes	60/50	IRI	+
Chen et al., 2021	USA	*N =* 106; doctors	M_age_ = 38.57, SD = 7.41	49/57	LKM	Waiting list	1.5 h 3 times/week × 8 weeks; no home practice	no	106/106	JSPE	+
Nadler et al., 2020	Canada	*N =* 275; Company employees	unclear	unclear	online workplace-based mindfulness training	Waiting list	144 ~ 480 min	no	275/102	MEIA-W	+
Niu, 2020	China	*N =* 800; College students	M_age_ =21.33, SD =2.05	402/398	Buddhist meditation intervention	Blank control	3 h once/week × 8 weeks	no	Not tell	IRI	+
Orosa-Duarte et al., 2021	Spain	*N =* 103; Students of Medicine, Psychology, Nursing, or Nutrition	M_age_ = 23, SD =4.16	unclear	mindfulness-based mobile app	Blank control	200 min of sessions	no	103/68	JSPE	+
Orosa-Duarte et al., 2021	Spain	*N =* 100; Students of Medicine, Psychology, Nursing, or Nutrition	M_age_ = 24, SD =4.16	unclear	MBSR	Blank control	2.5 h once/week × 8 weeks	yes	100/61	JSPE	−
Pérula-de Torres et al., 2021	Spain	*N =* 126; tutors and resident intern specialist	M_age_ = 41.61, SD = 12.61	unclear	MBSR	Blank control	2.5 h/session × 4 sessions	yes	126/75	JSPE	−
Pérula-de Torres et al., 2021	Spain	*N =* 102; tutors and resident intern specialist	M_age_ = 41.61, SD = 12.61	unclear	MBSR	Blank control	2.5 h/session × 8 sessions	yes	102/88	JSPE	+
Shapiro et al., 2011	USA	*N =* 30; Undergraduate students	18 ~ 24 years	unclear	MBSR	Waiting list	1.5 h once/week × 8 weeks	no	30/30	IRI	+
Shapiro et al., 1998	USA	*N =* 78; medical and premedical students	unclear	unclear	MBSR	Waiting list	2.5 h once/week × 7 weeks	yes	78/73	ECRS	+
Verweij et al., 2018	Netherlands	*N =* 153; residents from all medical, surgical and primary care disciplines	M_age_ = 31.2, SD = 4.6	18/130	MBSR	Waiting list	2.5 h once/week × 8 weeks	yes	153/138	JSPE	+
Wallmark et al., 2013	Sweden	*N =* 42; healthy people	Intervention: M_age_=32, SD=11; control: M_age_=35, SD=15,	63/26	Buddhist meditation intervention	Waiting list	75 mins once/week × 8 weeks	yes	42/42	IRI	+

N, number; SD, Standard Deviation; M_age_, Mean Age; M, male; F, female; MBET, Mindfulness-based empathy training; LKM, Loving-kindness Meditation training; MBSR, Mindfulness-Based Stress Reduction; MBCT, Mindfulness-Based Cognitive therapy; JSPE, Jefferson Scale of Physician Empathy; IRI, The Interpersonal Reactivity Index; ECRS, Empathy Construct Rating Scale; MEIA-W, Multidimensional Emotional Intelligence Assessment. Positive findings (+) indicate significantly greater improvements in the MBIs group compared to the control group, and negative findings (−) indicate no greater benefits of MBIs compared to the control group.

**Table 2:** Subgroup characteristics of the included studies.

**Reference**	**Occupation**	**Format of intervention**	**Intervention dose**	**Type of intervention**	**Home practice**
Asuero et al., 2014	Medical related majors	Offline	28 h	mindfulness-based psychoeducational intervention	no
Can Gür and Yilmaz, 2020	Medical related majors	Offline	16 h	MBET	yes
Centeno, 2020	Non-medical related majors	Offline	32 h	MBCT	yes
Chan et al., 2021	Non-medical related majors	Offline	16 h	MBCT	yes
Chen et al., 2021	Medical related majors	Offline	36 h	LKM	no
Nadler et al., 2020	Non-medical related majors	Online	144–480 min	Online Workplace-Based Mindfulness Training	no
Niu, 2020	Non-medical related majors	Offline	24 h	Buddhist meditation intervention	no
Orosa-Duarte et al., 2021	Medical related majors	Online	200 min	Mindfulness-Based Emotion Regulation	no
Orosa-Duarte et al., 2021	Medical related majors	Offline	20 h	MBSR	yes
Pérula-de Torres et al., 2021	Medical related majors	Offline	10 h	MBSR	yes
Pérula-de Torres et al., 2021	Medical related majors	Offline	20 h	MBSR	yes
Shapiro et al., 2011	Non-medical related majors	Offline	12 h	MBSR	no
Shapiro et al., 1998	Medical related majors	Offline	17.5 h	MBSR	yes
Verweij et al., 2018	Medical related majors	Offline	20 h	MBSR	yes
Wallmark et al., 2013	Non-medical related majors	Offline	10 h	Buddhist meditation intervention	yes

MBET, Mindfulness-based empathy training; LKM, loving-kindness meditation training; MBSR, Mindfulness-Based Stress Reduction; MBCT, mindfulness-based cognitive therapy.

Of the 13 studies (Shapiro et al., 1998, 2011; Wallmark et al., 2013; Asuero et al., 2014; Verweij et al., 2018; Can Gür and Yilmaz, 2020; Centeno, 2020; Nadler et al., 2020; Niu, 2020; Chan et al., 2021; Chen et al., 2021; Orosa-Duarte et al., 2021; Pérula-de Torres et al., 2021), 12 were randomized controlled studies and 1 was a quasi-experimental design (Centeno, 2020). A total of 2,135 individuals participated in the 13 studies, but gender ratios (Shapiro et al., 1998, 2011; Centeno, 2020; Nadler et al., 2020; Orosa-Duarte et al., 2021; Pérula-de Torres et al., 2021) and mean age (Shapiro et al., 1998, 2011; Nadler et al., 2020; Chen et al., 2021) were not confirmed in some studies. Study sample sizes were highly variable, ranging from 30 to 800.

Studies included diverse samples: undergraduate students (Shapiro et al., 1998, 2011; Can Gür and Yilmaz, 2020; Niu, 2020; Chan et al., 2021; Orosa-Duarte et al., 2021), healthcare professionals (Asuero et al., 2014; Verweij et al., 2018; Chen et al., 2021; Pérula-de Torres et al., 2021), company employees (Nadler et al., 2020), and Psychology majors (Centeno, 2020). Moreover, 7 out of the 13 studies focused on subjects in medical-related occupations (Shapiro et al., 1998; Asuero et al., 2014; Verweij et al., 2018; Can Gür and Yilmaz, 2020; Chen et al., 2021; Orosa-Duarte et al., 2021; Pérula-de Torres et al., 2021).

A range of intervention types were employed, and the most commonly used approach was MBSR (Shapiro et al., 1998, 2011; Verweij et al., 2018; Orosa-Duarte et al., 2021; Pérula-de Torres et al., 2021), two studies used MBCT (Centeno, 2020; Chan et al., 2021) and Buddhist meditation interventions (Wallmark et al., 2013; Niu, 2020), other studies used mindfulness-based psychoeducational intervention (Asuero et al., 2014), mindfulness-based emotion regulation (Orosa-Duarte et al., 2021), mindfulness-based empathy training (MBET) (Can Gür and Yilmaz, 2020), and loving–kindness meditation training (LKM) (Chen et al., 2021). The definitions of these intervention types were provided in the Supplementary materials 3.

Intervention cycle was generally 8 weeks, with 1 or 2 sessions per week of 60–150 min, but the total dose varies from 144 min to 32 h. The control group was the waiting list (Shapiro et al., 1998, 2011; Wallmark et al., 2013; Asuero et al., 2014; Verweij et al., 2018; Nadler et al., 2020; Niu, 2020; Chan et al., 2021; Chen et al., 2021) or blank control (Can Gür and Yilmaz, 2020; Centeno, 2020; Orosa-Duarte et al., 2021; Pérula-de Torres et al., 2021).

Empathy was evaluated using the Jefferson Empathy Scale (Asuero et al., 2014; Verweij et al., 2018; Can Gür and Yilmaz, 2020; Chen et al., 2021; Orosa-Duarte et al., 2021; Pérula-de Torres et al., 2021) and Interpersonal Reactivity Index scale (Shapiro et al., 2011; Wallmark et al., 2013; Centeno, 2020; Niu, 2020; Chan et al., 2021). Shapiro et al. (1998) used Empathy Construct Rating Scale (ECRS) for rating and Nadler et al. (2020) used Multidimensional Emotional Intelligence Assessment (MEIA-W). The former three scales are classical empathy scales that provide an overall level of empathy, and the empathy is only one variable in the latter one scale. However, what the four scales have in common is that higher scores indicate greater empathy.

The most frequently used mode of delivery was offline in an in-person way, which was used by 13 data (Shapiro et al., 1998, 2011; Wallmark et al., 2013; Asuero et al., 2014; Verweij et al., 2018; Can Gür and Yilmaz, 2020; Centeno, 2020; Niu, 2020; Chan et al., 2021; Chen et al., 2021; Orosa-Duarte et al., 2021; Pérula-de Torres et al., 2021). Two interventions were delivered online (Nadler et al., 2020; Orosa-Duarte et al., 2021). Subjects were asked to do home practice after class in 9 data (Shapiro et al., 1998, 2011; Wallmark et al., 2013; Verweij et al., 2018; Can Gür and Yilmaz, 2020; Centeno, 2020; Chan et al., 2021; Chen et al., 2021; Orosa-Duarte et al., 2021; Pérula-de Torres et al., 2021) and 6 data had no home practice (Shapiro et al., 2011; Asuero et al., 2014; Nadler et al., 2020; Chen et al., 2021; Orosa-Duarte et al., 2021).

### Quality appraisal of literature

**Figure 2:**
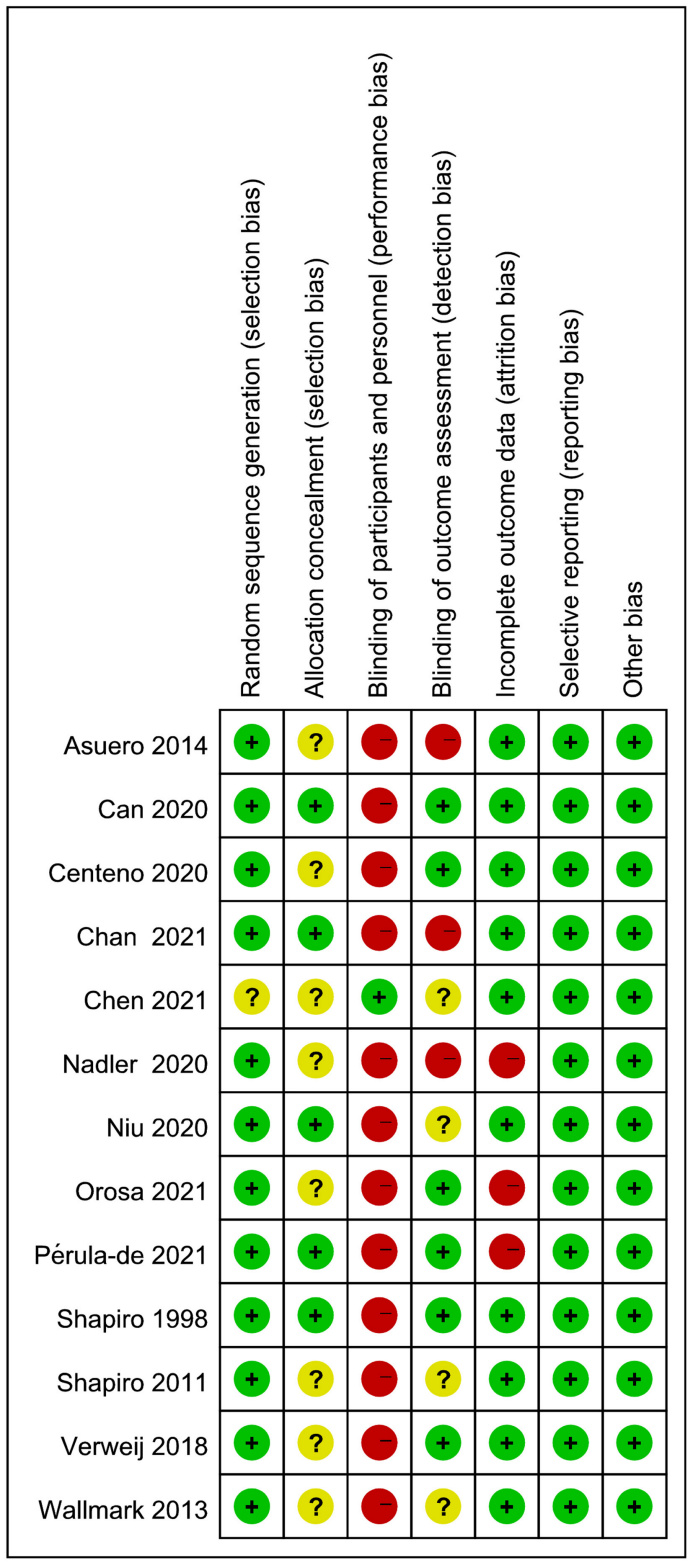
Risk of bias assessment across included studies.

The risk of bias assessment of the included literature is shown in Figure 2. The risk of bias in random sequence generation was generally low, and only 1 RCT does not specify or describe the randomization method used (Chen et al., 2021). We judged 8 studies that did not explicitly describe concealment of allocation (Shapiro et al., 2011; Wallmark et al., 2013; Asuero et al., 2014; Verweij et al., 2018; Centeno, 2020; Nadler et al., 2020; Chen et al., 2021; Orosa-Duarte et al., 2021), and the remaining 5 studies were judged to be at unclear risk of bias in this domain (Shapiro et al., 1998; Can Gür and Yilmaz, 2020; Niu, 2020; Chan et al., 2021; Pérula-de Torres et al., 2021). Owing to the specificity of intervention, only 1 study represented the methodology of blinding (Chen et al., 2021) and the remaining 12 studies were according to unsuccessful blinding and assessed at high risk. These studies were judged at high risk of bias because they claimed that assessors were not unsighted (Asuero et al., 2014; Nadler et al., 2020; Chan et al., 2021). Moreover, 6 studies were rated at low risk of bias (Shapiro et al., 1998; Verweij et al., 2018; Can Gür and Yilmaz, 2020; Centeno, 2020; Orosa-Duarte et al., 2021; Pérula-de Torres et al., 2021) and 4 were rated at unclear risk of bias in this domain (Shapiro et al., 2011; Wallmark et al., 2013; Niu, 2020; Chen et al., 2021). Three articles were rated as high risk in the incomplete outcome data owing to high shedding rates (Nadler et al., 2020; Chen et al., 2021; Orosa-Duarte et al., 2021), and the remaining 10 studies were judged to be at unclear risk of bias in this domain (Shapiro et al., 1998, 2011; Wallmark et al., 2013; Asuero et al., 2014; Verweij et al., 2018; Can Gür and Yilmaz, 2020; Centeno, 2020; Niu, 2020; Chan et al., 2021; Pérula-de Torres et al., 2021). All included articles were rated at low risk of bias in selective reporting and other bias.

### Effectiveness of MBIs

#### Outcomes: Empathy

**Figure 3:**
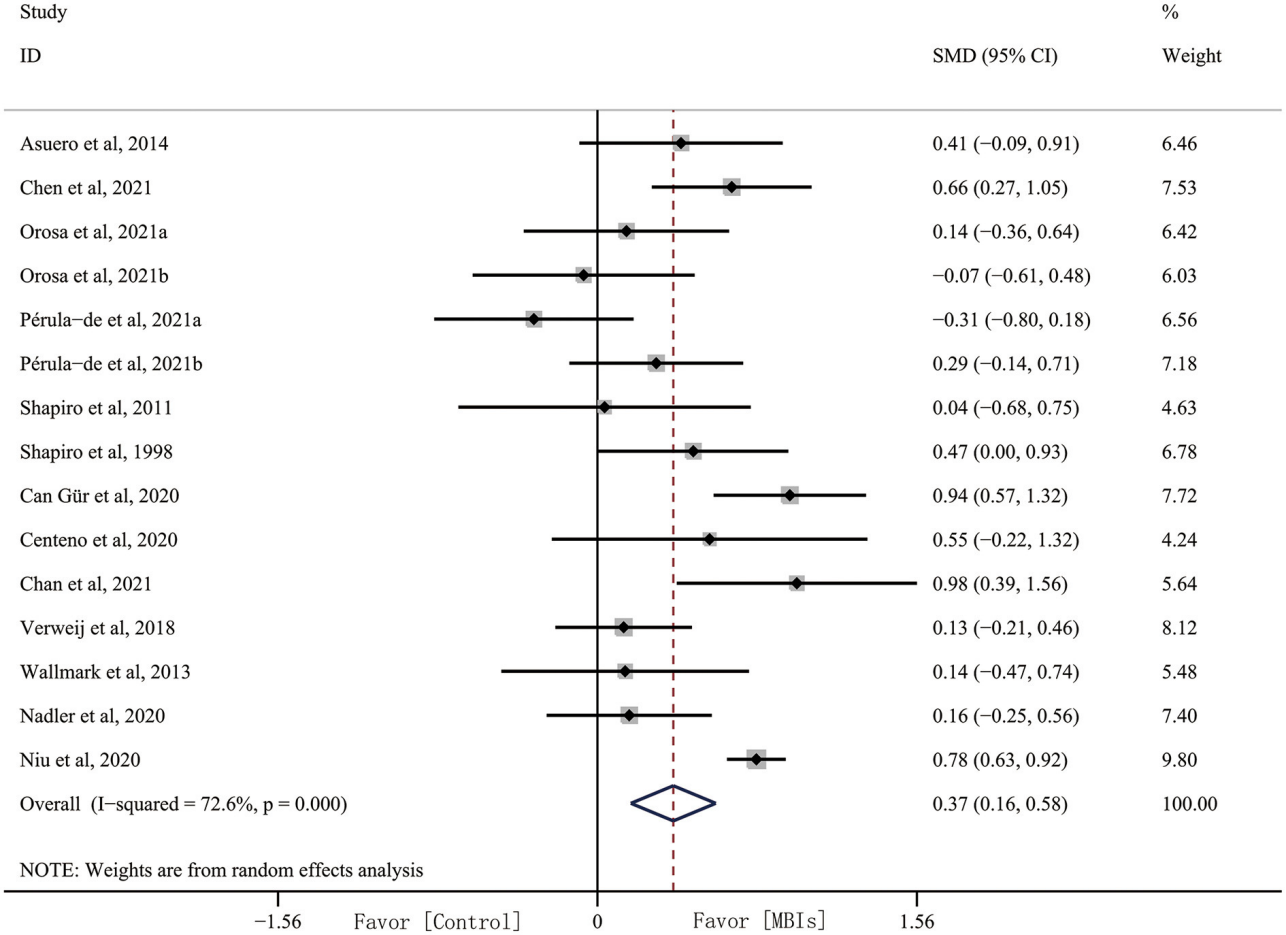
Mindfulness-based interventions (MBIs) effects on empathy.

Adequate data were available from 15 data for analysis. The results of the quantitative analysis showed that the effect size of MBIs on empathy was 0.372 (95% CI, 0.164–0.579, *p* = 0.001) with high heterogeneity (*I*^2^ = 51.01%, *p* = 0.001). This result indicated that MBIs have a significant effect on empathy (Figure 3). However, there was high heterogeneity, and we conducted subgroup analyses based on intervention dose, intervention format, intervention type, occupation, and home practice.

#### Subgroup analyses: Intervention dose

**Figure 4:**
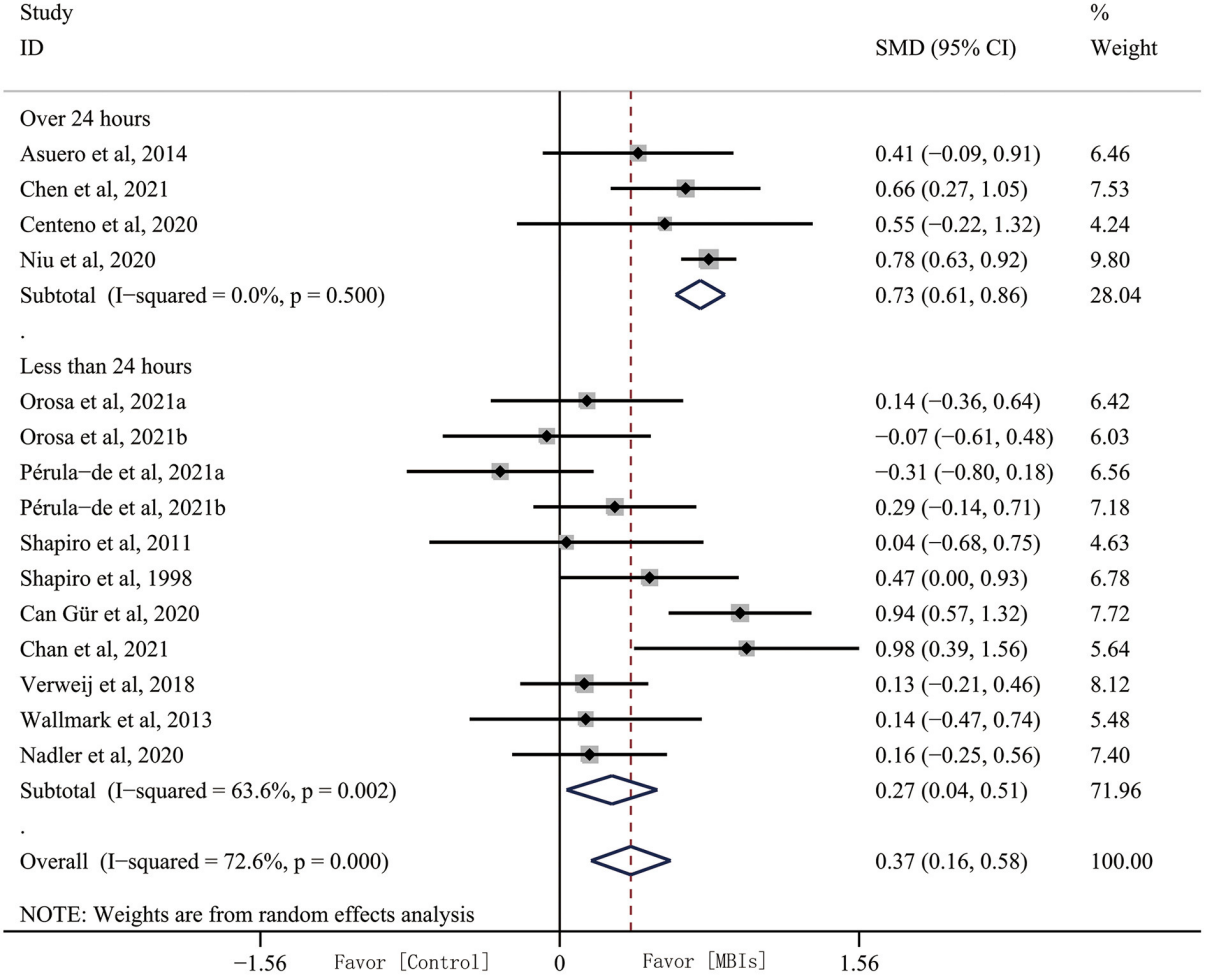
Subgroup analyses by intervention dose.

Intervention dose appears to be an important factor in treatment outcome, and we pooled the duration of the entire intervention cycle into a group with over 24 h and a group with under 24 h. Subgrouping analysis by intervention dose increased the effect size and decreased heterogeneity in the over 24 h subgroup (SMD, 0.735, 95% CI, 0.606–0.863, *p* = 0.001) with heterogeneity (*I*^2^ = 0.00%, *p* = 0.500). Effect size was decreased in the under 24 h group (SMD, 0.273, 95% CI, 0.036–0.510, *p* = 0.024) with heterogeneity (*I*^2^ = 63.6%, *p* = 0.002). Heterogeneity increased as well (Figure 4).

#### Subgroup analyses: Intervention format

**Figure 5:**
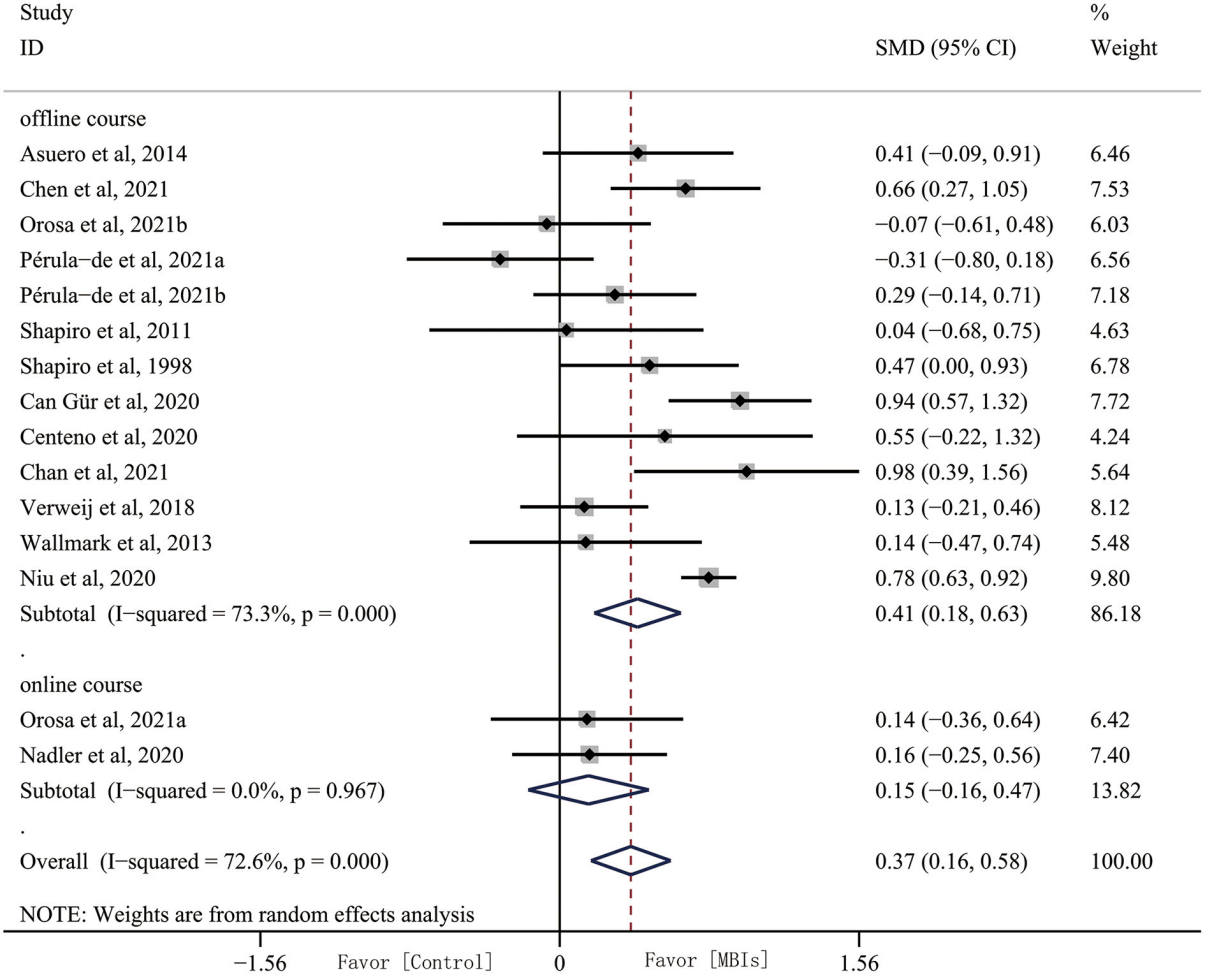
Subgroup analyses by format of intervention.

Most of the studies we included were conducted offline in an in-person way, and only two studies explored the effects of online MBIs interventions on empathy. Subgroup analyses found that MBIs are effective in improving subjects' empathy offline (SMD, 0.407, 95% CI, 0.181–0.632, *p* = 0.001) with heterogeneity (*I*^2^ = 44.87%, *p* = 0.001). However, effect size of online MBI intervention was reduced (SMD, 0.150, 95% CI, −0.165–0.465, *p* = 0.350) with heterogeneity (*I*^2^ = 0%, *p* = 0.967) (Figure 5).

#### Subgroup analyses: Intervention type

We compared the effects of different types of MBIs on empathy, and the results indicated that each type of intervention is able to improve empathy. However, the effects were different across interventions. MBET (SMD, 0.942, 95% CI, 0.569–1.315) and MBCT (SMD, 0.820, 95% CI, 0.352–1.288) were the most effective treatments, in which the effect sizes of both interventions are significantly higher than the total effect sizes. However, this conclusion is unreliable owing to the limited number of studies. Note that MBSR (SMD, 0.115, 95% CI, −0.099–0.329, *p* = 0.293) with heterogeneity (*I*^2^ = 20.5%, *p* = 0.279), which is one of the most used interventions, has a lower effect size compared with the total effect size (Supplementary Figure S1).

#### Subgroup analyses: Occupation

We divided the included subjects into two populations based on occupation: subjects with non-medical- and with medical-related occupations. Subgrouping analysis by occupation slightly increased the effect size in the non-medical-related occupations subgroup (SMD, 0.472, 95% CI, 0.136–0.809, *p* = 0.006) with heterogeneity (*I*^2^ = 67.9%, *p* = 0.008). The heterogeneity increased in medical-related occupation subjects (SMD, 0.314, 95% CI, 0.060–0.567, *p* = 0.015) with heterogeneity (*I*^2^ = 66.9%, *p* = 0.098). The results of the study indicated that MBIs are effective for empathy in non-medical- and medical-related occupation subjects (Supplementary Figure S2).

#### Subgroup analyses: Home practice

We explored the effect of home practice on MBI intervention. Subgroup analysis showed that the effect size increased slightly in the no home practice group (SMD, 0.424, 95% CI, 0.135–0.713, *p* = 0.006) with heterogeneity (*I*^2^ = 69.6%, *p* = 0.004). Neither heterogeneity nor effect size changed significantly in the home practice group (SMD, 0.341, 95% CI, 0.049–0.633, *p* = 0.022) with heterogeneity (*I*^2^ = 69.4%, *p* = 0.001) (Supplementary Figure S3).

### Publication bias and sensitivity analysis

**Figure 6:**
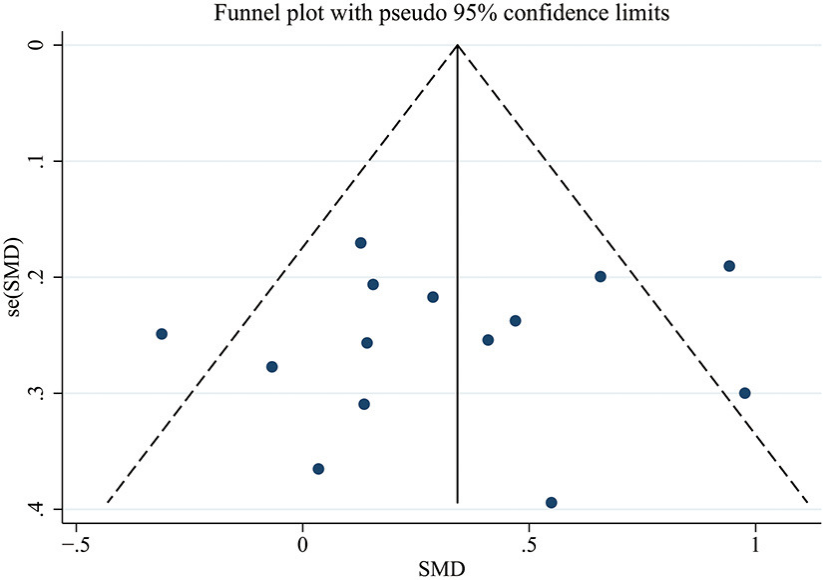
Publication bias funnel plots of standard errors and effect sizes of the included studies.

To test for publication bias in the literature included in the current study, a funnel plot was drawn (Figure 6) to show that the distribution of the studies was relatively symmetrical. Egger's test corroborated this finding (*p* = 0.655), indicating that our results were robust.

### Heterogeneity test and sensitivity analysis

We performed a heterogeneity test on the 15 data included in the current study (*I*^2^ = 51.01%, *p* = 0.001), and the results suggested strong heterogeneity. When literature comparisons were removed individually, heterogeneity did not change significantly. We performed a sensitivity analysis to find the reason for the strong heterogeneity. The results indicated that none of the studies had results that would have a considerable impact on the meta-analysis.

## Discussion

This study aims to investigate the effects of MBIs on empathy in a healthy population and the potential factors affecting the efficacy of MBIs. First, meta-analysis was performed on all MBI studies applied to increasing empathy in healthy populations. Compared with the control group, MBIs had a significant improvement in empathy in healthy adults, consistent with the results of Kreplin et al. (2018) and Luberto et al. (2018). Second, the results of the subgroup analysis showed that the intervention dose affects the effectiveness of the treatment, with better outcomes for interventions over 24 h. Each type of intervention was able to improve empathy, but the effects were different across interventions. Effect sizes of MBET and MBCT were significantly higher than the total effect sizes. Limited improvement was shown in MBIs for empathy in the online intervention group. Third, differences in the effectiveness of MBIs were not significant in the subgroup comparisons of occupation and home practice.

The mechanisms by which MBIs affect empathy are not fully understood. This situation can be explored from the perspectives of cognitive neuroscience and emotion regulation. Previous studies have shown that the anterior insula and anterior cingulate cortex (ACC) are the core networks of empathy (Bahji et al., 2021). Evidence based on neuroimaging studies has indicated that MBIs can cause specific brain changes associated with empathy. Hölzel et al. (2007) found that rostral ACC and dorsal medial prefrontal cortex (dmPFC) are activated in both hemispheres during Vipassana meditation. Imaging data showed that the body-mind training (IBMT) group has stronger activity in ACC compared with the same dose of relaxation training (Tang et al., 2009). Farb et al. (2013) found that during interoceptive to respiratory sensations, the MBSR group caused greater activation of the anterior insula than the waiting list and the coupling between dorsomedial PFC and posterior insula was also changed. Weisz and Cikara (2021) hold that emotion regulation also plays an important role in empathy, and enhanced emotional regulation is considered the basis for the effectiveness of mindfulness meditation (Tang et al., 2015). Positive effects of mindfulness meditation on emotion processing may include such aspects as reducing the disturbance of emotions by unpleasant stimuli (Froeliger et al., 2012), reducing difficulties in emotion regulation (Robins et al., 2012), and helping individuals return to an emotional baseline after responding to stressful stimuli (Goleman and Schwartz, 1976). Emotion may be the mediating factor that mediates MBIs to regulate empathy.

### Intervention dose

In a subgroup analysis of intervention dose, 4 studies had a total intervention dose of over 24 h and 11 studies had an intervention dose of under 24 h. Effect size of the long intervention group was significantly higher than that of the short intervention group, indicating that subjects in the former had better empathy outcome than those in the latter. We suspect that this result may be caused by the superimposed effect of MBI intervention, which requires a long period to achieve the desired effect. The result is consistent with Berry et al. (2018), who found that a minute of positive meditation develops empathy and increases helpful behavior toward strangers. Moreover, a three-month meditation practice based on thoughtful observation improved performance on empathy and even increased cortical volume in corresponding brain regions. Pérula-de Torres et al. (2021) compared the effectiveness of an abbreviated MBSR training program (2.5 h/session × 4 sessions) in relation to a standard training program (2.5 h/session × 8 sessions) on the levels of mindfulness, self-compassion, and self-perceived empathy in tutors and resident intern specialists. The results showed that only standard MBSR increased the level of empathy. The results of the subgroup analysis fill in the gap in the studies by Weisz and Cikara (2021), in which the effect of MBIs on empathy is strongly correlated with the dose of the intervention, with superior effects over 24 h.

### Intervention format

Kuhlmann et al. (2016) have argued that in-person MBI sessions are limited by the need of experienced therapists, delivery venues, participant schedules, and time-consuming characteristics. An increasing number of studies have explored the use of online MBI sessions in different fields (Champion et al., 2018; Sun et al., 2022). Orosa-Duarte et al. (2021) and Nadler et al. (2020) proposed the use of a cellphone app format for MBI sessions, and the results suggested that online MBI sessions had limited effect on improving the empathy. Given that only two studies were included in this analysis, additional research is needed to validate the effectiveness on empathy. Owing to convenience, effectiveness, and low cost, online MBI sessions provide a new way to promote MBI sessions (Gál et al., 2021). Future studies should focus considerably on online MBI sessions to further determine their positive effects on empathy.

### Intervention types

There is a rich variety of intervention types of MBIs, and MBSR was the most applied type of MBIs in the included studies (Shapiro et al., 1998, 2011; Verweij et al., 2018; Orosa-Duarte et al., 2021; Pérula-de Torres et al., 2021). However, the effect sizes for MBSR were lower than the overall effect sizes, suggesting that we can consider other types of MBIs in practice. The results of the subgroup analysis showed differences in the effects of different interventions. The possible reason is the limited number of articles, resulting in insufficient motivation for the analysis. In the future, additional articles of different intervention types on empathy may change this conclusion.

### Occupation

Owing to the unique nature of their professions, doctors, nurses, medical students, and other medical-related professionals are faced with considerable stressful situations in their works or studies, including wounds, illnesses, and even death (Williams and Stickley, 2010; Laughey et al., 2021; Pérula-de Torres et al., 2021). Accumulated stress takes a huge toll on their empathic abilities. Imparied empathy may likewise lead to a lack of effective communication between physicians and patients, thereby possibly leading to doctor–patient disputes and conflicts. The results of the subgroup analysis showed that MBIs are effective in improving empathy for medical- and non-medical-related professional subjects, and there are no significant differences between the two groups. Such results suggest that MBIs are worth promoting in a wide range of occupations.

### Home practice

After the in-person MBI sessions, subjects were asked to complete certain homework practice to improve the intervention effect of the MBI sessions. However, the results of our subgroup analysis showed that the homework practice did not affect the outcome of the intervention. This outcome appears to contradict the results of the intervention dose subgroup analysis; we speculate that this result may be caused by poor compliance and lack of professional coaching. Accordingly, improving compliance with home practice is also a research direction worth investigating.

### Strengths and limitations

For the strengths of this study, the current research is the first meta-analysis on the effectiveness of MBIs on empathy. Compared with previous studies, we included more articles to explore the factors affecting the effectiveness of MBIs by conducting subgroup analysis, thereby providing some references for the generalized application of MBIs. We employed rigorous meta-analytical techniques and assessment, such as sensitivity analyses and assessment of publication bias.

However, there are still some limitations in this study. Firstly, 9 out of 13 studies did not conduct follow-up visit, so we were unable to determine the long-term effects of MBIs on empathy. Future studies could be designed with follow-up programs to explore the long-term effects of MBIs for empathy. In addition, this study did not include gray literature, thereby potentially affecting the results of the meta-analysis. Secondly, the population of this study focused on healthy adults, which may be a limitation for promoting MBIs. Thirdly, as 8 out of 13 included articles did not inform about the sex ratio, and 3 out of 13 did not inform about age (Table 1). The data about gender and age included in the current study were not enough to support subgroup analysis, and subsequent studies can do some exploration based on these issues. Lastly, we only included 13 studies and the quantity of members was moderately minimal. Hence, more articles of high quality and large sample sizes are needed in the future to explore the intervention effects of different populations and different intervention protocols.

### Future directions

Previous studies have found that many diseases had empathy disorders, such as psychiatric disorders (Decety and Moriguchi, 2007; Bragado-Jimenez and Taylor, 2012; Schreiter et al., 2013) and chronic pain (Sohn et al., 2016; De Tommaso et al., 2019; Ma et al., 2020; Mu et al., 2021; Zhang et al., 2021). It would be meaningful to see if the extension of MBIs to these subjects could improve their empathy. To our knowledge, there are limited studies on the use of MBIs in the elderly population, and exploring the effects in different populations may be a potential direction for future research.

## Conclusions

The present study confirmed the effect of MBIs on the improvement of empathic ability in healthy populations. Subgroup analyses revealed that intervention dose, formats, and types are key factors influencing intervention effects. Future research should markedly focus on large-sample, rigorously designed experiments to explore the long-term effects of MBIs on empathy and to elucidate the underlying mechanisms of MBIs. We are optimistic that the findings of this study will provide evidence for teachers and physicians to design programs to prevent empathy reduction and identify alternative gaps for future research.

## Data availability statement

The original contributions presented in the study are included in the article/Supplementary material, further inquiries can be directed to the corresponding author.

## Author contributions

Conceptualization: Z-YH, Y-RW, and Y-LW. Methodology: Y-RW. Software: JS. Validation: Z-YH and Y-LW. Formal analysis: Z-HY. Investigation: Y-RW and Z-HY. Resources and project administration: Z-YH. Data curation: Y-TL. Writing-original draft preparation and writing-review and editing: Y-RW and Z-YH. Visualization: Y-FW. Supervision: Y-YL and Y-LW. All authors have read and agreed to the published version of the manuscript.

## Funding

This research was funded by the Guangdong Hopson-Pearl River Education Development Foundation, Grant Number H20190116202012724.

## Conflict of interest

The authors declare that the research was conducted in the absence of any commercial or financial relationships that could be construed as a potential conflict of interest.

## Publisher's note

All claims expressed in this article are solely those of the authors and do not necessarily represent those of their affiliated organizations, or those of the publisher, the editors and the reviewers. Any product that may be evaluated in this article, or claim that may be made by its manufacturer, is not guaranteed or endorsed by the publisher.
