# Supplementary material for: Effectiveness of mindfulness-based interventions on empathy: A meta-analysis
Source: Front Psychol. 2022 Oct 20;13:992575. doi: 10.3389/fpsyg.2022.992575 (PMC9632989; doi:10.3389/fpsyg.2022.992575)
Supplement: Supplementary file 2 [file Table_2.DOCX]

Pubmed:

("Theory of mind"[tw] OR ToM[tw] OR "mentali*"[tw] OR "role taking"[tw] OR "perspective taking"[tw] OR empathy[mh]) AND ("Mindfulness" [Mesh] or "Meditation" [Mesh] or "mindfulness*"[tw] or "mindfulness-based" [tw] or MBSR[tw] or MBCT[tw] or "M-BCT" [tw] or meditation[tw] or "meditat*" [tw] or Vipassana[tw] or satipaṭṭhāna[tw] or anapanasati[tw] or Zen[tw] or Pranayama[tw] or Sudarshan[tw] or Kriya[tw] or zazen[tw] or shambhala[tw] or "buddhis*" [tw])

WOS:

(ALL= Mindfulness OR ALL= " Meditation " OR ALL= mindfulness OR ALL= “mindfulness-based” OR ALL= MBSR OR ALL= MBCT OR ALL= “M-BCT” OR ALL= “meditat*” OR ALL= Vipassana OR ALL= satipaṭṭhāna OR ALL= anapanasati OR ALL= Zen OR ALL= Pranayama OR ALL= Sudarshan OR ALL= Kriya OR ALL= zazen OR ALL= shambhala OR ALL= “buddhis*”) AND (ALL= “Theory of mind” OR ALL= ToM OR ALL= “mentali*” OR ALL= “role taking” OR ALL= “perspective taking” OR ALL=empathy)

Cochrane:

(Mindfulness OR " Meditation " OR mindfulness OR “mindfulness-based” OR MBSR OR MBCT OR “M-BCT” OR “meditat*” OR Vipassana OR satipaṭṭhāna OR anapanasati OR Zen OR Pranayama OR Sudarshan OR Kriya OR zazen OR shambhala OR “buddhis*”) AND (“Theory of mind” OR ToM OR “mentali*” OR “role taking” OR “perspective taking” OR empathy)

Embase:

(Mindfulness OR " Meditation " OR mindfulness OR “mindfulness-based” OR MBSR OR MBCT OR “M-BCT” OR “meditat*” OR Vipassana OR satipaṭṭhāna OR anapanasati OR Zen OR Pranayama OR Sudarshan OR Kriya OR zazen OR shambhala OR “buddhis*”) AND (“Theory of mind” OR ToM OR “mentali*” OR “role taking” OR “perspective taking” OR empathy)

CNKI:

SU= (共情 + 移情 + 通情）AND SU=（正念 + 正念疗法 + 正念训练 + 正念冥想 + 正念减压 + 正念认知）
