# Supplementary material for: Effectiveness of mindfulness-based interventions on empathy: A meta-analysis
Source: Front Psychol. 2022 Oct 20;13:992575. doi: 10.3389/fpsyg.2022.992575 (PMC9632989; doi:10.3389/fpsyg.2022.992575)
Supplement: Supplementary file 3 [file Table_3.DOCX]

**Mindfulness-based stress reduction (MBSR)**: MBSR is a well-established mindfulness training that has shown to reduce stress, depression, and anxiety. MBSR teaches individuals to observe situations and thoughts in a nonjudgmental, nonreactive, and accepting manner. MBSR provides training in formal mindfulness practices, including body scan, sitting meditation, and yoga. MBSR seeks to change the individual's relationship with stressful thoughts and events by decreasing emotional reactivity and enhancing cognitive appraisal. The standard MBSR curriculum is conducted in an 8-week structured group format, which includes weekly 2.5-hour group sessions in addition to a 6-hour daylong retreat. (Khoury et al., 2015)

**Mindfulness-based cognitive therapy (MBCT)**: Mindfulness-based cognitive therapy (MBCT) is an 8-week group training that combines mindfulness meditation techniques with elements of cognitive-behavioral therapy. MBCT teaches participants to recognize and disengage from maladaptive automatic cognitive patterns, and to develop a nonjudgmental and compassionate attitude toward their own cognitions and feelings. MBCT has been demonstrated to be effective in reducing relapse. (Cladder-Micus et al., 2018)

**Mindfulness-based psychoeducational intervention:** The program was designed to enhance the physician-patient relationship through reflective practices that help the practitioner explore the domains of control and meaning in the clinical encounter. The course is based on 3 techniques: mindfulness meditation, narrative medicine, and appreciative inquiry. (Krasner et al., 2009)

**Mindfulness-based emotion regulation:** It is based on an online application for Android and iOs. It proposes a guided training program through eight stages. Each stage is divided into three sections called Listening, Practicing, and Integration into everyday life. The content of the program was developed by accredited MBSR teachers. The app provides short videos with explanations about the fundamentals of mindfulness, self-compassion, and the physiological stress reaction, as well as audio segments that guide practices of mindfulness, for a total of more than 200 min of sessions. It has been designed following the international recommendations for the development of apps (MARS guide). (Orosa-Duarte et al., 2021)

**Buddhist meditation interventions:** The Buddhist meditation approach is composed of the concentration on the breath or mindfulness of body, sensations, mind and mental phenomena or calm abiding, which can lead to the subsequent relaxation. (Amarasekera and Chang, 2019)

**Mindfulness-based empathy training (MBET):** The MBET was a structured weekly program that included a variety of experiences based on the improvement of mindfulness abilities. The didactic expression technique, role-playing technique, model learning technique, and homework were used. Formal practices in-class and at-home included mindfulness breathing, body screening meditation, thought awareness, and dried grape meditation. Informal practices included mindfulness in daily life activities, communication, automatic pilot, and staying present. Each education focalized on a certain topic planned on raising mindful awareness of empathy. (Can Gür and Yilmaz, 2020)

**Loving–kindness meditation training (LKM):** The meaning of LKM is to care for oneself and all creatures. Its purpose is to cultivate unconditional feelings of love, kindness, and acceptance. In the practice of LKM, the practitioner directs kindness first to himself, then to loved ones, acquaintances, strangers, and, finally, to all sentient beings. LKM has many effects and can be practiced at any time in a variety of positions. (Chen et al., 2021)

Reference:

Amarasekera, A. T. and Chang, D. (2019). Buddhist meditation for vascular function: A narrative review. *Integr Med Res,* 8**,** 252-256. doi:10.1016/j.imr.2019.11.002

Can Gür, G. and Yilmaz, E. (2020). The effects of mindfulness-based empathy training on empathy and aged discrimination in nursing students: A randomised controlled trial. *Complement Ther Clin Pract,* 39**,** 101140. doi:10.1016/j.ctcp.2020.101140

Chen, H., Liu, C., Cao, X., Hong, B., Huang, D. H., Liu, C. Y., et al. (2021). Effects of Loving-Kindness Meditation on Doctors' Mindfulness, Empathy, and Communication Skills. *Int J Environ Res Public Health,* 18. doi:10.3390/ijerph18084033

Cladder-Micus, M. B., Speckens, A. E. M., Vrijsen, J. N., Ar, T. D., Becker, E. S. and Spijker, J. (2018). Mindfulness-based cognitive therapy for patients with chronic, treatment-resistant depression: A pragmatic randomized controlled trial. *Depress Anxiety,* 35**,** 914-924. doi:10.1002/da.22788

Khoury, B., Sharma, M., Rush, S. E. and Fournier, C. (2015). Mindfulness-based stress reduction for healthy individuals: A meta-analysis. *J Psychosom Res,* 78**,** 519-28. doi:10.1016/j.jpsychores.2015.03.009

Krasner, M. S., Epstein, R. M., Beckman, H., Suchman, A. L., Chapman, B., Mooney, C. J., et al. (2009). Association of an educational program in mindful communication with burnout, empathy, and attitudes among primary care physicians. *Jama,* 302**,** 1284-93. doi:10.1001/jama.2009.1384

Orosa-Duarte, Á., Mediavilla, R., Muñoz-Sanjose, A., Palao, Á., Garde, J., López-Herrero, V., et al. (2021). Mindfulness-based mobile app reduces anxiety and increases self-compassion in healthcare students: A randomised controlled trial. *Med Teach,* 43**,** 686-693. doi:10.1080/0142159x.2021.1887835
