# Supplementary figures and images for: Effectiveness of mindfulness-based interventions on empathy: A meta-analysis
Source: Front Psychol. 2022 Oct 20;13:992575. doi: 10.3389/fpsyg.2022.992575 (PMC9632989; doi:10.3389/fpsyg.2022.992575)

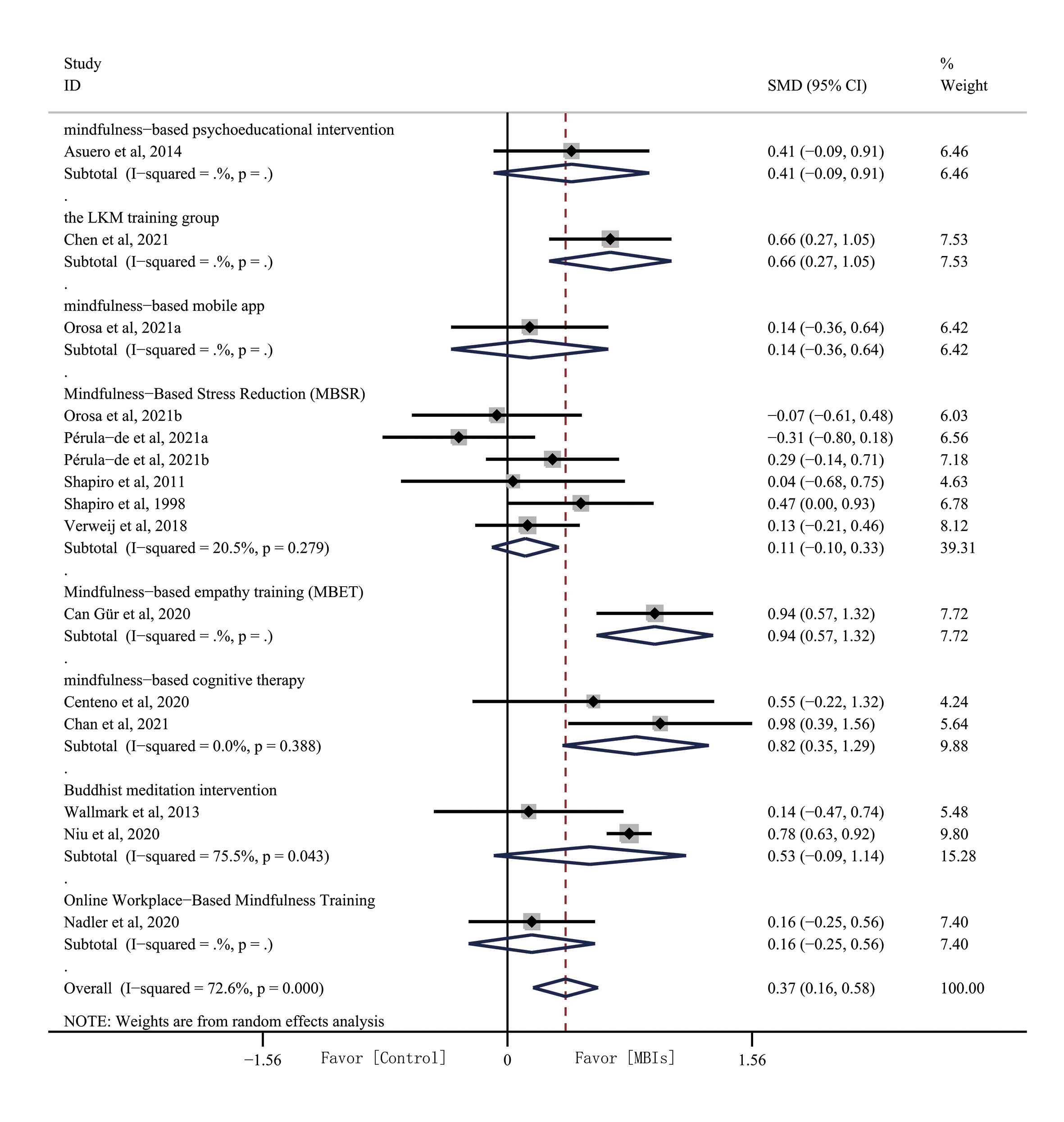

Supplement: Supplementary file 4 [file Image_1.TIF]

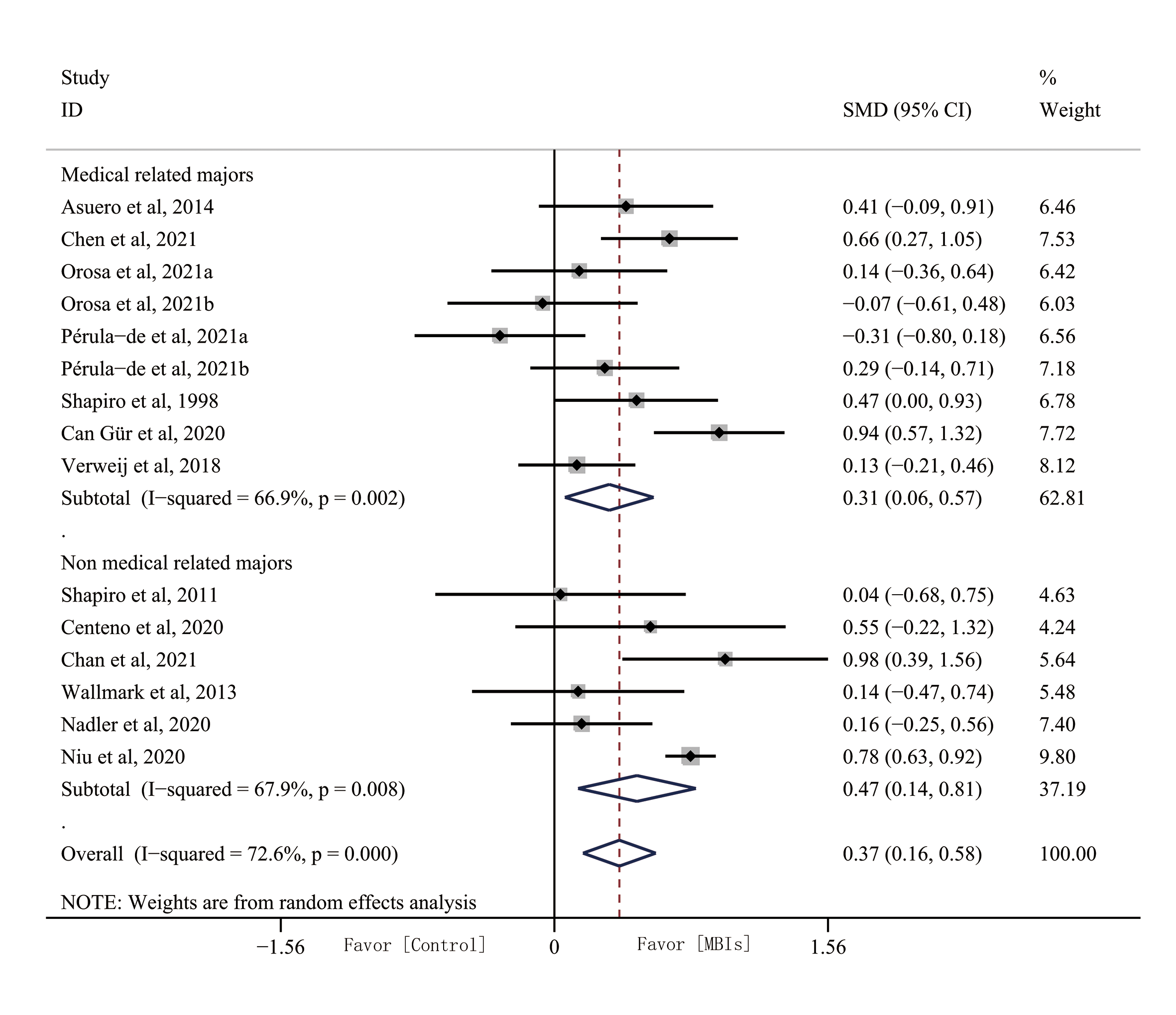

Supplement: Supplementary file 5 [file Image_2.TIF]

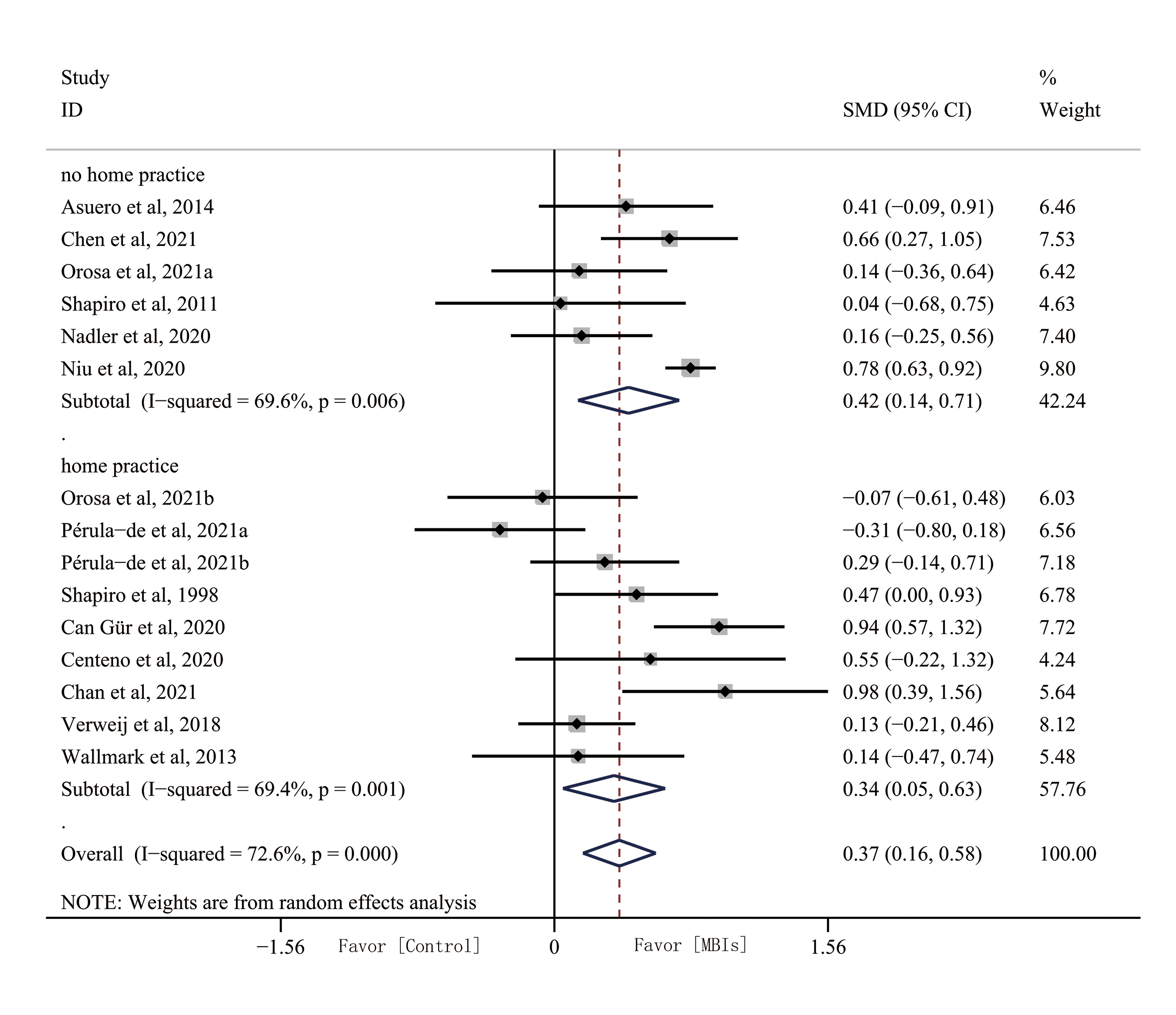

Supplement: Supplementary file 6 [file Image_3.TIF]
